# Diet, Genetics, and Disease: A Focus on the Middle East and North Africa Region

**DOI:** 10.1155/2012/109037

**Published:** 2012-03-29

**Authors:** Akl C. Fahed, Abdul-Karim M. El-Hage-Sleiman, Theresa I. Farhat, Georges M. Nemer

**Affiliations:** ^1^Department of Genetics, Harvard Medical School, 77 Avenue Louis Pasteur, Boston, MA 02115, USA; ^2^Department of Biochemistry and Molecular Genetics, American University of Beirut, Beirut 1107 2020, Lebanon

## Abstract

The Middle East and North Africa (MENA) region suffers a drastic change from a traditional diet to an industrialized diet. This has led to an unparalleled increase in the prevalence of chronic diseases. This review discusses the role of nutritional genomics, or the dietary signature, in these dietary and disease changes in the MENA. The diet-genetics-disease relation is discussed in detail. Selected disease categories in the MENA are discussed starting with a review of their epidemiology in the different MENA countries, followed by an examination of the known genetic factors that have been reported in the disease discussed, whether inside or outside the MENA. Several diet-genetics-disease relationships in the MENA may be contributing to the increased prevalence of civilization disorders of metabolism and micronutrient deficiencies. Future research in the field of nutritional genomics in the MENA is needed to better define these relationships.

## 1. Introduction

Over the past few decades, the MENA has been witnessing significant changes in food habits paralleled by an important preponderance of metabolite-related diseases. In a region whose traditional diet is known to be healthy due to high vegetable proteins, fibers, minerals, and vitamins with low content of unfavorable food products, the “industrialization/westernization of the diet” is a well-studied and documented phenomenon [1–3]. The MENA has been losing its traditional diet which was distinguished by its diversity and richness in raw foods, proteins, and multivitamins, in the favor of a more industrial diet which consists of increased preprocessed foods, sugars, fats, alcohol, animal products, saturated- and trans-fatty acids, and relatively less vitamins and minerals with decreased consumption of milk, fruits, and vegetables [4]. A big part of this change is attributed to the lifestyle changes and globalization with the invasion of western fast food to the MENA countries. Dietary choices, minimum physical activity, religious habits, consumer ignorance, high population growth rates, economic factors, and lack of both protection laws and food fortification programs are other critical factors that influence the nutritional status in the region [5]. These changes in dietary and lifestyle patterns contribute to an increase in the rates of micronutrients deficiencies, diet-related chronic diseases, and obesity in all groups of the population in the region [5]. Due to this grave impact on chronic diseases, diet became a target of public health initiatives that aim at restoring the traditional diet of MENA countries in order to improve health conditions in their populations [6–8]. The epidemiology of diet-related diseases in the MENA region and background information on the diet-genetics-disease interaction, followed by nutrigenomic examples on diet-related diseases in the MENA region, will be discussed for the first time in this paper.

## 2. Epidemiology of Diet-Related Diseases in the MENA

**Figure 1:**
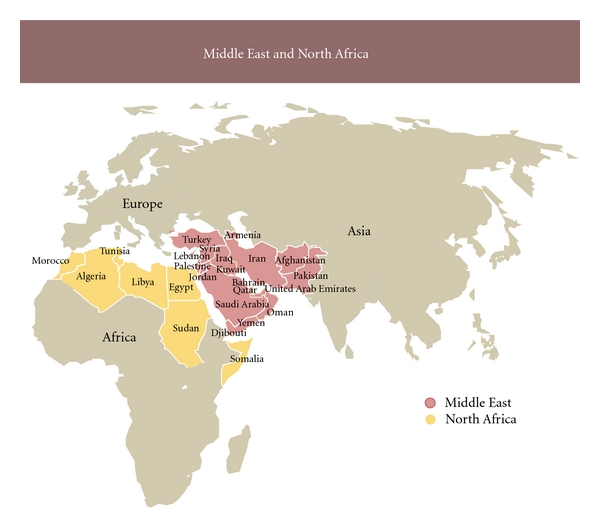
Map of the Middle East and North Africa (MENA) region. The MENA region includes countries such as Algeria, Armenia, and Turkey, that are not members of the WHO Eastern Mediterranean Region (EMR) that is referred to in the literature.

We review the numbers and trends for selected chronic metabolic diseases and micronutrient deficiencies in the different countries of the MENA where data are available (Figure 1). Given the lack of nationwide data, prevalence rates are commonly reported as estimations [9, 10]. Since 1982, the need for “direct evidence of a secular [increase]” in diseases has been established, prospecting an association with “acculturation” of traditional or rural populations [11]. Still, the numbers show alarming trends for cardiovascular diseases and metabolic disorders, namely, insulin resistance, adiposity, dyslipidemias, and atherosclerosis. Likewise, micronutrient deficiencies (MNDs) have been heavily studied recently due to their crucial contribution to the global burden of many chronic diseases.

**Figure 2:**
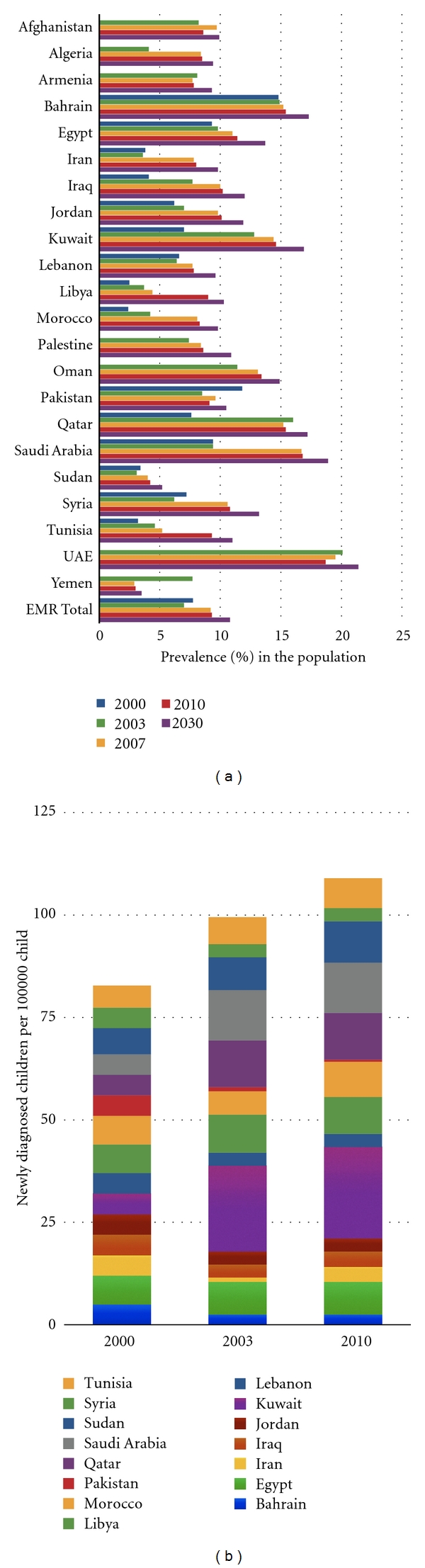
Increasing prevalence of diabetes mellitus in the MENA. (a) General increase in reported prevalence (%) of Type 2 Diabetes Mellitus in the MENA between the years 2000 and 2010. Numbers are reported as approximated by the International Diabetes Federation [12–15]. The expected 2-fold increase for the year 2030 is approximated based on demographic parameters, without accounting for changes in age strata or other risk factors [16]. (b) Overall growth in annual incidence (per 100,000) of Type 1 diabetes mellitus in children younger than 14 years old in the MENA. Numbers are estimations by the International Diabetes Federation based on various years between 1986 and 2000 [12–15].

**Figure 3:**
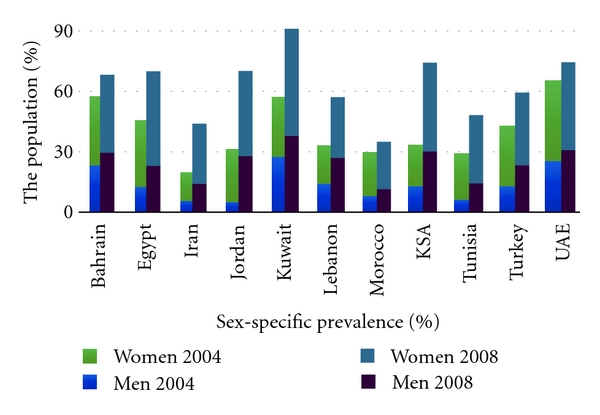
Increasing prevalence of obesity in the MENA. Prevalence (%) of obesity increased in both men and women in countries of the MENA between 2004 and 2008. Numbers are WHO estimates in World Health Statistics of 2005 and 2011. Totals of men and women are integrated for purposes of comparative illustration and do not represent adjusted arithmetic total prevalences. Obesity was defined as body mass index (BMI) ≥30 Kg/m^2^. Obesity data about Jordanian men in 2004 are not available, but prevalence was estimated to be less than that of women [17, 18]. KSA: Kingdom of Saudi Arabia; UAE: United Arab Emirates.

**Figure 4:**
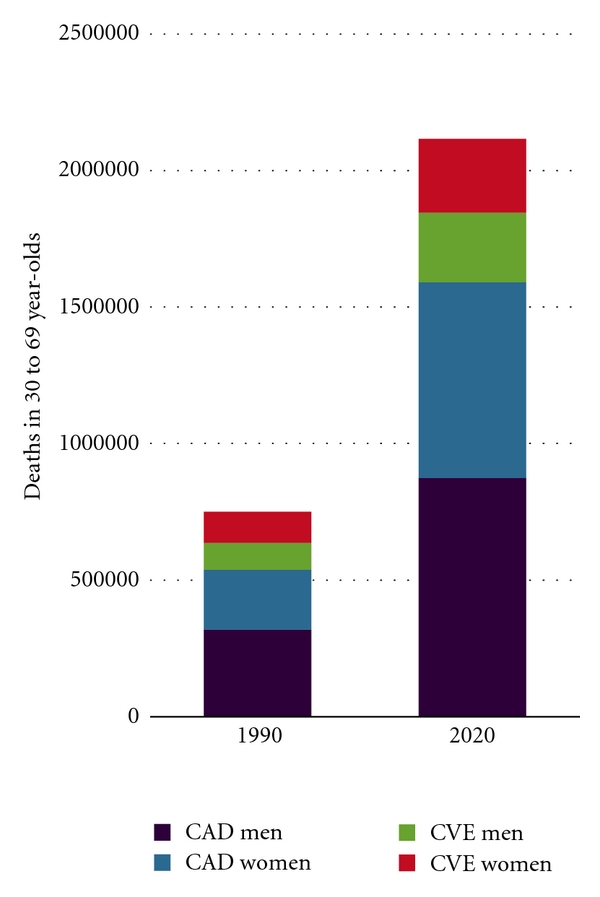
Expected overall increase in mortality due to cardiovascular diseases in the MENA [19]. CAD: Coronary Artery Disease; CVE: Cerebrovascular Event.

The region is witnessing an “explosion” of Type 2 Diabetes Mellitus (T2DM) according to the International Diabetes Foundation (IDF) (Figure 2(a)) with five countries of the MENA ranking among the top ten diabetic worldwide [15]. Similarly, the IDF reports an increase in the incidence of Type 1 DM in children of the MENA over the past decade (Figure 2(b)). The World Health Statistics of the WHO show a sharp rise in the prevalence of obesity (Figure 3), which is the most powerful and easily reported risk marker and hence the sole epidemiological WHO indicator for cardiovascular and metabolic comorbidities. In one epidemiological study, cardiovascular mortality in the MENA has been estimated to triple from 1990 to 2020 (Figure 4).  Table 1 summarizes rates of cardiovascular diseases and two major comorbid risk factors, hypertension and the metabolic syndrome, with wide variations across countries of the MENA.

In the other category of diseases, deficiencies in vitamins and minerals affect particularly children and women of childbearing age. MNDs impair physical and mental development of children, exacerbate infections and chronic diseases, and impact morbidity and mortality. Most countries of the MENA have widespread MNDs, yet countries of the Gulf Cooperation Council, Iran, and Tunisia have moderate levels of MNDs [20]. Among MNDs, iron deficiency is the most prevalent nutritional problem in the MENA [21]. Its prevalence in the region varies from 17 to 70% among preschoolers, 14 to 42% among adolescents, and 11 to more than 40% among women of childbearing age. Severe iron deficiency is a direct cause of anemia. Nonnutritional genetic anemias are known to be relatively common in the region but will not be tackled because they are not affected by dietary factors. Nutritional deficiencies other than iron, such as folic acid, vitamin B12, and vitamin C, are also prevalent in some countries of the region but data are scarce [20]. The Middle Eastern and South Asian regions have the highest rates of Vitamin D Deficiency (VDD) worldwide [22]. The prevalence ranges of VDD in the MENA are 46–83% for adolescents and adults, and 50–62% among veiled women [5]. It reaches up to 70% in Iran [23] and 80% in Saudi Arabia [24].  Table 2 shows prevalence of VDD and iron deficiency in countries of the MENA where data are available.

## 3. The Dietary Signature on the Genome

**Figure 5:**
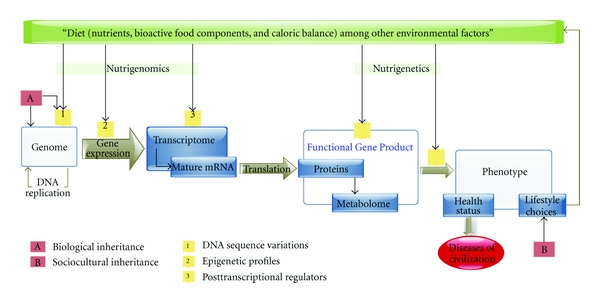
The dietary signature. The biologically inherited DNA genome accumulates DNA sequence variations over generations. Epigenetic profiles determine which parts of it are to be transcribed. Once transcribed to RNA, it matures into different mature RNA outcomes depending on the post-transcriptional regulators. Among the effects of the environment on DNA sequence variations, epigenetic profiles, and post-transcriptional regulators, the effect of diet is studied in nutrigenomics. After translation, and under the impact of dietary status surrounding the primarily translated proteome, the final set of functional proteins, activated pathways, and subsequent metabolites constitutes the functional Gene Product. The gene product is only potentially functional towards a certain phenotypic outcome. The downstream end result of health status depends greatly on what nutrients are fed into the systemic machine of gene products. The functional gene product is the end-point in nutrigenomics and the starting point in nutrigenetics. It is a marker of the phenotypic outcome: expression of disease and prognosis. Phenotype may dictate the lifestyle choices available to a certain individual, including taste preferences, which are also delineated by culturally inherited customs and habits. In their turn, lifestyle choices including dietary habits determine environmental exposures. Furthermore, civilization diseases have been hidden for a long period of time due to the sociocultural inheritance of adequately evolved matching lifestyle preferences and diet choices that have been masking a biologically inherited limited gene pool. The genes being in *status quo*, in presence of a nutritional transition, the rates of civilization diseases are on the rise because of the loss of the protective adequacy of the diet. This highlights the presence of hidden genes, the phenotypic expression of which can be masked by a specific nutritional state, such as that corresponding to the Mediterranean diet, as more increasingly being recommended recently in the literature. However this cannot be answered if sequence variations and specific SNPs affecting nutritional needs are not tested for in the specific populations.

The effect of food on gene function is the focus of nutritional genomics, an emerging field of study that focuses on the molecular, cellular, and systemic levels of this effect [25, 26]. The abundance of calories, macro- and micronutrients, and bioactive food elements constitutes the nutritional environment that alters the genome, the epigenome, the posttranscriptional regulation, and the posttranslational modifications, leading to a variety of metabolic functional gene-products, as shown in Figure 5. The nutritional environment channels the function of gene-products into certain pathways, preferring certain biological activities over others and resulting in the final phenotypic outcome and health-disease status. Correspondingly, two disciplines of nutritional genomics are entertained: nutrigenomics and nutrigenetics. The former started as a focus on the effect of nutrients on gene expression, while the latter, yet a different field of study, emerged in search of approaches to alter the clinical manifestations of certain rare diseases, such as certain inborn errors of metabolism, via personalized diet. The link between them is the functional gene product. It is the end-point in nutrigenomics and the starting point in nutrigenetics. Other conventional terminologies relevant to the diet-genetics-disease relation will also be treated in the following section, prior to discussion of the dietary signature on the genome in the MENA.

(1) The *genome sequence* of DNA base pairs dictates the primary genetic profile and hence gene function. DNA sequence variants—gene variants and single nucleotide polymorphisms (SNPs)—designate an alteration of gene structure with or without functional changes that might or might not lead to a complex gene-function relationship depicted in different diseases [27]. The epigenome, an emerging concept in genetic research, is a set of nongenetic factors, such as diet, that change the expressed gene outcome without affecting the structure of the DNA *per se*.

(2)* Nutrigenomics* considers environmental factors of alimentary source that may disrupt the DNA sequence in peptide-coding and in promoter regions, affecting the gene product. Other environmental nutrigenomic factors include abundance of macro and micronutrient components of the diet, presence of other bioactive food elements, and caloric content. Under- or overnutrition in the maternal environment sets epigenetic programming mechanisms via energetic control of function and oxidation. Through regulation of many biological functions including mitochondrial activity, cellular stress, inflammation, and telomere shortening, the dietary signature starts when epigenetic mechanisms induce or limit the risk to disease [28]. Possible levels of expression of a certain gene lie in a range of disease susceptibility that is determined by epigenetic mechanisms. These mechanisms are dictated by the functional profile of the cell, which obeys its nutritional state and reflects the nutritional environment.

(3) Although sustainability of *epigenetic programming* along life span is not well understood [28], two temporally distinct profiles may be distinguished. First, the basal epigenome is determined early on in life. Depending on the basal expressivity of DNA, it behaves like a permanently edited version of the genome. Accordingly, increasing evidence of trans-generational inheritance of epigenetics was found in mice [29] through the effect of grandmaternal nutrition on grandchildren during gamete stage, throughout the mother's fetal stage [30]. Second, later in life, similar mechanisms affect gene expressivity in response to temporary environmental factors, resulting in a short-lived epigenetic profile. These changes are mainly due to interference of nutrients and bioactive food components with transcription factor conformations [26]. This signature serves as a means for the organism to receive information about its nutritional environment in order for the cells to execute appropriate modifications on the profile of expressed genes [31]. The nutrigenomic signature is not well studied in humans yet; however obvious importance is due to its impact on gene expression, chronic diseases susceptibility, and health status of future generations [31–33].

(4) Regulators of *posttranscriptional modifications* affect alternative RNA splicing which gives rise to different mature mRNA isoforms. Alternative splicing is as highly prevalent as in 35 to 59% of human genes [34]. Post-trancriptional regulators, such as microRNAs and their coacting and counteracting proteins, are part of the RNA and protein pools [35]. They are hence influenced by epigenetic and metabolic factors as well [26, 36].

(5)* Proteomics* is the study of the protein pool in the organism, as an integral part of the cellular function. On the other hand, the metabolome designates the structure, the localization, the post-translational modifications, and the functions of proteins and metabolites along with their interactions in the organism [37]. It defines the current metabolic state and active intracellular pathways in the organism (Figure 5). The functional gene products comprise all the potentially functional molecules and pathways, whether currently active or not, that result from a certain genome-epigenome combination, leading to a certain range of possible phenotypic outcomes, rather than a clearly defined health status.

(6) *Nutrigenetics* is a quite different approach that emerged when dietary interventions were able to successfully alter the course of certain diseases. The basic principle considers how the same dietary environment can result in different phenotypic outcomes of health or disease in metabolizers with different functional gene-products or programmed phenotypes [38]. The concept is similar to how individuals possess different phenotypes as drug-metabolizers. The study of genetic variations affecting nutrient metabolism, from digestion to detoxification, can decipher ambiguities in the diet-disease relationship [38]. However, the challenge lies in the ability of researchers to describe the processes through which the dietary environment imposes itself to precipitate metabolic disorders.

(7) Finally, hypotheses of *Thrifty Profile*, namely thrifty genes and thrifty phenotypes, offer explanations for etiology, predisposition, and rising prevalence of DM and obesity. Early life dietary habits foretell the basal appetite control and cellular nutritional needs through psychological and molecular habituations [28]. Thrifty genes that enable survival during periods of food shortages may have been conserved over generations under the selection pressure of under-nutrition [39]. Thrifty phenotypes may be due to early nutritional challenges that enhance nutrients-saving mechanisms in the growing individual, leading to excessive storage later on and increased risk of metabolic disorders [40]. Both models have not gathered enough evidence apiece; however combined they provide a fertile base for further nutritional genomic research.

An example of a phenotype that has evolved accordingly is taste preferences and ability to digest, absorb, and appropriately respond to nutrients [41]. Genes for taste receptors, among other proteins that handle the metabolism of different nutrients, have been extensively studied. In an extensive review by Garcia-Bailo et al., an important aspect of the dietary signature is addressed: the genetic variations that affect dietary habits and food choices, with an emphasis on their effects on the nutritional environment and the health outcome [41].

 Given the rise in multifactorial diseases, nutrigenetics started to involve public health research, hinting at personalized dietary recommendations for prevention of civilization diseases many years before clinical manifestations arise [31]. Adequacy of the general dietary recommendations to the ancient nature of our genes is becoming increasingly dubious. The human genome, as we know it, was sculpted throughout 2 million years of evolution under the diets of our hunters-gatherers ancestors [42]. Later on, the available food choices changed since the introduction of agriculture, but too rapidly for the ancestral stone-age genome to keep up with. This fast nutritional transition revealed evolutionary origins of obesity and diabetes among other civilization epidemics [43]. The experiment-based advancement of dietary recommendations during the past 25 years showed a convergence towards what looks more like a Paleolithic hunter-gatherer diet [44]. Despite low compliance to recommended diets and increasing industrialization of actual dietary habits, personalized and ancestral dietary recommendations still seem promising.

## 4. Nutritional Genomics in the MENA

 Diet, genetics, and disease are linked in many ways as could be shown in Figure 5. The MENA is a region that has been witnessing simultaneously a dietary change and a worsening prevalence of chronic diseases. Because of this, nutritional genomics research in such a region can improve our understanding of this rapid change in disease prevalence and shed light on the genomic effects of this dietary transition in the region. Nutritional genomics research in the MENA is minimal. To our knowledge, this is the first review on the topic in the region. We aim to collate studies in MENA countries that discuss any aspect of the dietary signature that we discussed in Figure 5. We approach that using examples of common diseases with rising prevalence in the MENA. The paper discusses two categories of diseases: (1) civilization disorders of metabolism (cardiovascular diseases and metabolic risk factors), and (2) micronutrient deficiencies (MNDs).

We also look at other populations where nutritional genomics research in these disease categories was done and discuss how it applies to our region with recommendations for future research on MENA populations.

## 5. Diet-Related Civilization Disorders of Metabolism in the MENA Region

 Populations of the MENA belong to a unique genetic pool because of the mixture of ethnicities with horizontal mixing of populations throughout history, the high rate of consanguineous marriages within subpopulations, and the geography of the states making up the region (Figure 1). Nevertheless, wide prospective population studies on the effects of polymorphisms on such disorders of metabolism in the MENA are still lacking [25]. Based on literature reports on other populations, a large set of genes and DNA sequence variants are potentially culpable of the rise of metabolic disorders under the effect of industrialized diets. In this part of the paper, we present numerous polymorphisms that predispose to metabolic disorders including T2DM, obesity, dyslipidemias, atherosclerosis, cardiovascular events, and hypertension. The civilization disorders have common pathophysiologies and risk factors of metabolism and, since interrelated and comorbid, will accordingly be treated as one major health outcome in the following discussion about genetic entities common to the different disorders, under the effect of diet.

The *Brain-Derived Neurotrophic Factor* (*BDNF*) is important for energy balance in mice and for regulation of stress response in humans (OMIM 113505). Polymorphisms in this gene are associated with obesity and all subtypes of psychological eating disorders in Europeans (NCBI 627). Recently, three-way association was identified between hoarding behavior of obsessive-compulsive disorder, obesity, and the Val/Val genotype of *BDNF *in the Valine (Val) to Methionine (Met) amino acid change at position 66 (Val66Met) in Caucasians [45]. The suggested evolutionary mechanism for this complex relationship between gene, psychopathology, and body weight is the conservation of a thrifty gene, once an old survival strategy.

 Control of fetal appetite was recently shown to be a function of the *Fat Mass- and Obesity-Associated *(*FTO*) gene expression [28] which codes a nuclear oxygenase that affects tissue lipid metabolism [46] (NCBI 79068) and depends on energy balance during development [28]. A strong relation links *FTO* SNPs to higher risk of obesity and T2DM in many international studies (OMIM 610966) [47, 48].

*Transcription Factor 7-Like 2* (*TCF7L2*) gene codes a transcription factor involved in blood glucose homeostasis. The rs7903146 variant association to T2DM varies greatly over ethnicities (OMIM 602228). However in Palestinians, this SNP (114758349C > T) increases the risk for T2DM, and homozygotes are affected at younger age [49].

*Calpain 10* (*CAPN10*), which codes a calcium-dependent cysteine protease, is being increasingly studied for its role in T2DM (NCBI 11132). SNP-44 of *CAPN10* has significant association with T2DM and total cholesterol in Gaza [50], while only UCSNP-19 SNP and haplotype-111 are proven to be high risks for T2DM in Tunisia [51].

 The association of T2DM with polymorphisms of the *Angiotensin Converting Enzy*me (*ACE*) and the *Methylene Tetrahydrofolate Reductase* (*MTHFR*) is not well proven [52]. However data in Tunisians suggest synergistic action of the *ACE* Insertion/Deletion (I/D) dimorphism with the *MTHFR *C677T SNP on risk of T2DM [52]. Fairly common, *ACE* D and *MTHFR *677T alleles are, respectively, present in around 77 and 27% of Moroccans [53]. Nevertheless, *ACE* DD genotype in Tunisians is associated with higher ACE activity and might become a useful clinical marker for CAD risk assessment of acute myocardial infarction [52, 54]. In Lebanon, *ACE* D allele and age, combined, are associated with higher risk for hypertension [54]. Also, Lebanese with *MTHFR *C677T turned out to be more susceptible to diabetic nephropathy than Bahrainis with the same SNP [55]. The SNP cannot constitute an independent risk factor in Arabs [56]. Its effect is presumably due to high homocysteine levels and hence must be evaluated depending on dietary and ethnic backgrounds [55].

 In genes encoding the G protein-coupled Beta-2- and 3-Adrenergic Receptors (*ADRB2, ADRB3)*, evolutionary selection of specific alleles exists in Africans, Asians, and Europeans. *ADRB2 *Glu27 and Gln27 are, respectively, factors of exercise-dependent obesity risk and metabolic syndrome susceptibility (OMIM 109690). Glu/Glu and Glu/Gln can independently predict severe Coronary Artery Disease (CAD) in Saudi Arabs [57]. However *ADRB3* Trp64Arg SNP is a CAD predictor only in presence of other risk factors in Arabs, but not an independent one [57]. ADRB3 is mainly located in adipose tissues causing easier weight gain and earlier T2DM onset in Trp64Arg individuals in several populations [58] (NCBI 155) (OMIM 109691).

*Peroxisome Proliferator-Activated Receptor Gamma* (*PPARG*) genes encode nuclear receptors and regulators of adipocyte differentiation and possibly lipid metabolism and insulin sensitivity (OMIM 601487). Pro12Ala isoform of *PPARG2* seems to activate transcription less effectively and carry less morbidity. Carriers of a Pro12Ala polymorphism may have a weaker BMI correlation to *amount* of dietary fat when compared to Pro homozygotes [59], while response to *quality* of dietary fat is greater in terms of BMI, lipid profile, and fasting insulin levels [60, 61]. However these associations were not found for many of the studied populations (OMIM 601487).

Apolipoproteins (APOs) are involved in lipid metabolism. *APOE *polymorphisms have been heavily studied. In the *APOE *G219T SNP, TT individuals have prolonged postprandial lipemia [62]. *Apo E* has three major isoforms, E2, E3, or E4. *APOE *E4 individuals may be protected effectively by lower dietary fat intake [63] while non-E4 individuals have minimal to no benefit from dietary intervention on lipid profile [64]. In Iranians, E2 allele was associated with lower total cholesterol levels [65]. However, despite correlation between APOE2 and LDL subfraction profiles in healthy Arabs, no similar association was found in Arabs with CAD [66]. *APOE *E2, E3, and E4 carriers constitute approximately 11, 79, and 10% of Moroccans, respectively [53].

 Moreover, mutations in *Lipoprotein Lipase* (*LPL*), which is crucial for receptor-mediated lipoprotein uptake, drastically affect lipoprotein metabolism disorders (NCBI 4023). In Saudi Arab population however, lack of association between *LPL *polymorphisms and CAD was noticed [56]. Strong evidence exists for Hepatic Lipase (*LIPC*) C514T homozygotes. They have more atherogenic lipid profile in response to dietary fat in addition to impaired adaptation to higher animal fat with higher cardiovascular diseases risk [67, 68].

 Finally, the Paraoxonase (*PON1*) gene encodes for an anti-atherosclerotic esterase which capacitates high-density lipoproteins to prevent lipoprotein oxidation. Gln192Arg and Leu55Met are two common polymorphisms of *PON1 *that modulate PON1 activity in the serum, which predicts the architecture of apolipoprotein, lipoprotein, and lipid levels [69]. In the late 1990s, PON1 status, including genotype and serum activity levels, has been proven to predict cardiovascular risk much better than genotype alone [70]. However, in Turkish subjects, there was no consistent association between the polymorphisms and the lipid levels [71]. An individual's polymorphism might be suggestive of a high risk while his dietary signature is making the actual PON1 activity favorable, that is, low risk. This interaction between diet and genes can hinder the significance of genetic screening, and enhance the relevance of proteomics and metabolomics. The lesson learned from the *PON1* role in cardiovascular disease is of utmost relevance. Functional genomic analysis is required for adequate risk assessment; an individual may be screened for all known polymorphisms of *PON1*, but still not be assigned a risk category for cardiovascular disease [72].

 Discrepancies between genotype and function impose limitations on genetic screening. Similarly for most of the polymorphisms presented previously, the degree to which genetic screening can be helpful in decision-making is controversial. More activity correlation studies are needed to examine the “penetrance” of polymorphisms. Also, insufficient nutrigenomic and proteomic evidence may be misleading [117]. Hence, further multidisciplinary studies, with coordination between laboratories, will be needed to decide which gene/polymorphism would be worth screening in a particular population.

 Further multidisciplinary nutritional genomics research is needed for more specific targeted individualized advising and therapy. However, given the current lack of adequate understanding of the genetic etiologies of civilization diseases and wide-scale regional genetic screening studies, reversal of dietary changes is rendered the simplest available measure to control the metabolic epidemic of civilization diseases in the MENA.

## 6. Micronutrient Deficiencies (MNDs) in the MENA Region

 Micronutrients (vitamins and minerals) are required throughout life, in minute amounts in the human body, to function as cofactors of enzymes or as structural components of proteins, or to maintain genome stability, among other physiological roles [118]. Both their excess and deficiency may cause DNA damage, alter growth and development, contribute to a wide array of chronic diseases, and jeopardize health [118]. MNDs are highly prevalent in MENA countries as was established earlier. Deficiencies in iodine, iron, and vitamin A are very important MNDs in terms of prevalence and potential threat to public health worldwide; however relevant gene-diet interaction has not been sufficiently studied in the MENA. This section will thus be restricted to the following MNDs of particular interest in diet-genetics-disease interaction: vitamin D, calcium, iron, folate, and vitamins C, E, B6, and B12.

### 6.1. Vitamin D Deficiency (VDD)

**Figure 6:**
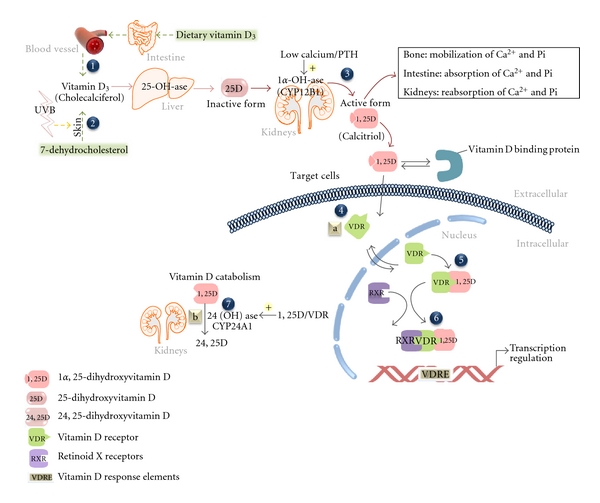
Vitamin D pathway and sites of interaction with dietary factors. Cutaneous or dietary vitamin D is hydroxylated in the liver to form 25-hydroxyvitamin D (1,2) and in the kidney to form 1*α*,25-dihydroxyvitamin D (3). 1*α*,25-dihydroxyvitamin D binds to VDR (4), the 1*α*,25-dihydroxyvitamin D ligand promotes VDR-RXR heterodimerization (5), and the complex binds to VDRE to mediate transcriptional regulation of target genes (6). The concept of gene-diet interaction is described in the vitamin D pathway by the different polymorphisms in the VDR gene (a) and the dietary regulation of CYP24A1 enzyme (b).

Vitamin D is a fat-soluble vitamin, with two forms, one present in a narrow range of foods (D2) and another formed under the skin when exposed to the ultraviolet B (UVB) light fraction of sunlight (D3); both are activated by the liver and kidneys [119] (Figure 6). Prolonged VDD can result in rickets in young children and osteoporosis and fractures in adults [120]. Recently, low vitamin D levels have been associated with increased risk of hypertension, cardiovascular diseases [121], cancer [122], diabetes [123], musculoskeletal and immunity disorders, and infectious diseases [124].

Despite the sunny climate, the MENA has a highly prevalent VDD across all age groups, with the highest rate of rickets worldwide [22]. The main reasons are limited sun exposure and low dietary vitamin D intake, along with frequent pregnancies, short breastfeeding periods [125], skin pigmentation [126], body mass index [127], religious practices [128], and educational levels [129].

In addition to nutritional and social factors of VDD, genetic factors also play an important role and are depicted on the metabolic pathway of vitamin D shown in Figure 6. Genetic variations predisposing to VDD are related to Vitamin D Receptor (*VDR*) polymorphisms at intron 8 (BsmI) and exon 2 (FokI) [130]. The Fok1 polymorphism (C>T) in the translation initiation site creates an upstream initiation codon and a three amino acids longer molecule in the f allele compared to the F allele [131] which gives a more transcriptionally active VDR [132] leading to the tolerance to low vitamin D levels observed in Egyptian FF homozygotes [130]. This suggests possible evolutionary adaption to dietary intake or lifestyle changes. Only FF homozygote children have increased calcium absorption and bone mineral density [131]. Conversely, the decreased calcium absorption linked to the f allele was correlated with an increase in colon cancer risk only when calcium dietary intake is low [133]. FF genotype seems hence more advantageous than ff genotype. Paradoxically, FF (shorter VDR) was correlated with rickets unlike ff (longer VDR) in Turks and Egyptians. Thus, further studies are needed to understand the complex genetics and risks of rickets. *VDR* B allele also predisposes to VDD since Egyptian B homozygotes had severe rickets [130]. In other studies in the Middle East, high vitamin D doses were needed to treat patients with rickets [134]. Thus, the unexpected high prevalence of VDD in the MENA could be linked to VDR polymorphisms.

Moreover, a G>A polymorphism at position −3731 of the cdx-2 (Caudal-Type Homeobox Transcription Factor) binding element on the *VDR* gene promoter is another genetic variant of *VDR* that affects calcium absorption. The Cdx2 promoter A allele (cdx-A) binds cdx2 more strongly and has a greater transcriptional activity compared to the cdx-G allele. Thus, the A allele may increase intestinal *VDR* expression, subsequently enhancing calcium intestinal absorption and increasing bone mineral density. The differential expression of *VDR* shows how genetic differences influence the body response to nutrients [135].

Bioactive food components may exert an effect on gene expression and enzyme activity, subsequently decreasing disease risk. For example, 4′,5,7-Trihydroxyisoflavone (genistein), a soy component, is a genome-protective nutrient. It inhibits the activity of CYP24A1 (Figure 6) and thus 1,25(OH)_2_D degradation, increasing VDR stability and the half-life and biological effects of vitamin D [136]. Folate can also inhibit this activity by increasing methylation of the promoter of *CYP24A1*. Also, addition of vitamin D and calcium to the western diet significantly decreases the incidence of colon cancer [133].

### 6.2. Iron Deficiency

Iron (Fe) is an essential mineral needed in small amounts mainly for the production of hemoglobin and utilization of oxygen among other vital functions. Iron deficiency is a common MND mostly caused by low intake of iron, blood loss, and parasitic infections [137]. Iron absorption is enhanced by vitamin C, low pH, and heme iron and hindered by bioactive vegetables components (polyphones, tannins, phytates) and calcium [138].

Genetic factors that contribute to iron deficiency, in addition to the dietary intake, are underscored by the ability of many individuals to maintain normal iron levels despite low iron dietary intake. Nutritional iron deficiency and genetic iron deficiency have been experimentally distinguished in mice. Hephaestin (Heph) is a multicopper oxidase that allows iron basolateral surface export. Sex-linked anemia (*Sla*) mice bearing a deletion in *Heph* gene compared to control mice showed different responses to diet; *Sla* mice had duodenal iron accumulation and low plasma iron [139]. Additionally, mutations in the genes of human hemochromatosis protein (*HFE*) and its interacting protein beta-2 microglobulin (*B2M*), which play an important role in iron metabolism, cause murine iron deficiency [140].

### 6.3. Folate Deficiency

Folic acid is a water soluble B vitamin of exclusive dietary origin. It provides the one-carbon metabolism with its main coenzyme form, tetrahydrofolate (THF). A key enzyme herein, methylenetetrahydrofolate reductase (MTHFR), catalyzes vitamin B12-dependent conversion of homocysteine to methionine, a precursor of S-adenosylmethionine (SAM), a methyl donor to DNA [141]. Thus, folate deficiency results in hyperhomocysteinemia (HHC), a risk factor for CAD [142].

*MTHFR *C677T is prevalent in 4% of Pakistanis [32], 2% of Yemenite Jews, 10% of Muslim Arab Israelis, and 11% of Lebanese [151]. It leads to a thermolabile MTHFR, which precipitates HHC in low folate states. This gene-environment combination is a risk factor for cardiovascular diseases [152], neural tube defects, and other chronic diseases [153].

C677T is a genetic variation that affects individual dietary requirements because it makes prevention of folate-related diseases that require higher folate intake. Korean C homozygotes develop HHC only with low folate levels, while T homozygotes have HHC even with normal folate levels [154]. Surprisingly however, the hyperhomocysteinemia, low folate levels, and increased cardiovascular risk observed in Indian Asians compared to European whites were not attributed to the *MTHFR* 677T variant [155]. Studies specific to the MENA are therefore needed because of possible variations from other regions.

The example of MTHFR and folate also underscores the genome-epigenome interplay. The TT genotype alters DNA methylation and gene expression in peripheral blood mononuclear cells only in folate deficient patients [156]. Paradoxically, the same polymorphism is inversely associated with and hence has a protective role against colorectal cancer (CRC). CRC risk decreases with adequate methionine intake, which leads to an increased formation of SAM and a negative feedback inhibition of MTHFR activity. However, the protective roles of MTHFR mutation and methionine dietary intake require an adequate dietary folate intake [157]. These findings suggest that inadequate folate intake puts carriers of particular genetic variants at higher risk of cancer. Thus personalized dietary interventions might be beneficial in reducing cancer risks.

Serum vitamin B12 deficiency is highly prevalent in Iran [158]. Thus, low folate and vitamin B12 levels can be inversely linked to the hyperhomocysteinemia observed in this population. Importantly, high prevalence of CAD was observed in a Turkish population with low plasma folate despite low plasma cholesterol concentrations [159]. It remains to determine the link between hyperhomocysteinemia, low folate intake, CAD risk, and MTHFR variants, to explain this prevalence in Turkey and possibly in other countries of the MENA region. This would add folate supplementation as a possible treatment for hyperhomocysteinemia. Other studies have also reported the possible contribution of the level of pyridoxal phosphate (PLP or vitamin B6) to hyperhomocysteinemia and vascular disease; low PLP levels were observed in individuals with the homozygote TT genotype compared to healthy individuals from some countries of the MENA [160, 161]. These data corroborate the various dietary signatures on the specific genetic profile and show that other mechanisms, enzymes, and vitamins should be examined as well.

### 6.4. Vitamin B12 Deficiency

Like folate deficiency, vitamin B12 (cobalamin) deficiency can affect establishment of the disease depending on the genetic background. A common genetic variant is detected in Methionine Synthase Reductase (MTRR), an important enzyme for maintaining Methionine Synthase in its active state. The polymorphism is an A66G substitution resulting in an Ile22Met residue. The homozygous genotype was associated with an increased risk of neural tube defects (NTDs) when combined with low vitamin B12 levels. Vitamin B12 deficiency is highly prevalent among Iranian women of childbearing age [158]. However polymorphisms in both *MTHFR* and *MTRR* increase NTDs risk [162].

### 6.5. Vitamins C and E Deficiencies

As antioxidants, vitamins C and E have an important function in the diet-gene interaction. Glutathione S-transferases (GSTs) transfer glutathione to different substrates. A common deletion of *GSTM1* gene, a deletion polymorphism in *GSTT1*, and an A313G polymorphism of *GSTP1* result, respectively, in a nonfunctional genotype, loss of enzyme activity, and altered activity of the *GST* isoforms. The GST enzymes were found protective against serum ascorbic acid deficiency when vitamin C consumption is low, since GST null genotypes with low vitamin C intake had an increased serum ascorbic acid deficiency risk [163]. The Hp1 and Hp2 polymorphisms in the hemoglobin-binding protein haptoglobin (Hp) were also studied in vitamin C deficiency. Unlike Hp1 carriers, Hp2 homozygotes had lowest serum vitamin C concentrations when dietary vitamin C intake is insufficient. Thus, Hp1 has a greater antioxidant capacity preventing hemoglobin-iron-related vitamin C oxidation and depletion [164].

A protective role for vitamin E against atherosclerosis, cancer, and neurodegenerative diseases has also been reported. Polymorphisms in the proteins involved in vitamin E metabolism lead to differential vitamin E uptake and response among individuals, and subsequently different disease risk [165]. Dietary vitamin E intake also influences the body mass index (BMI) and risk of obesity via modifying genetic variants of SIRT1 (sirtuin protein family of nicotinamide-adenine-dinucleotide- (NAD+)-dependent histone deacetylases) [166].

Well-studied gene-diet interactions are also critical in the pathophysiology of cancer. Given the evidence that VDR polymorphisms, MTHFR genotype, and DNA methylation in a low calcium or folate intake are associated with an increased cancer risk, the dietary signature greatly influences carcinogenesis. Significant dietary factors include antioxidants such as vitamin C, carotenoids, lycopene, tocopherols (vitamin E), and many other micronutrients present in fruits and vegetables.

Being one of the leading causes of death worldwide as well as in the MENA, cancer has been extensively studied, and the diet-genetics-cancer interaction is currently being thoroughly investigated for each and every one of the involved micronutrients. So, the effect of the diet-genetics interaction on carcinogenesis will not be dwelled upon in this paper.

The aforementioned studies collectively depict the interaction between diet, genetic variability, and disease. Genetic variants might affect gene expression patterns and epigenetic events resulting in differential body responses to diet. However, a small individualized nutritional intervention that is well studied to provide the needed concentrations of micronutrients can influence genetic variants to decrease disease risk. Thus, nutrigenetics is a tool for choosing the appropriate diet according to the individual's genetic makeup.

## 7. A Call for Nutritional Genomics Research in the MENA

All previous data support the crosstalk between diet and genome. Differential responses to dietary components among individuals are determined by genetic factors. The deleterious effects of some genotypes can be circumvented by an increased intake of particular nutrients to overcome the genetic susceptibility, which opens the horizon for personalized diet. In turn, nutrients might affect genome, gene expression, and phenotype. Although hard and complex, it is worthwhile to identify the genes that predispose individuals to chronic diseases and the nutrients that regulate their expressions to modify personal risks and to prevent, mitigate, or treat diseases. Studying on an individual basis the interactions between diet and genetics could help select appropriate diet to optimize health status. Indeed, the picture becomes more complicated when lifestyle, behavioral, and other environmental factors interfere with the diet-genetics interaction.

 Of note is the unique ethnic combination of the region's native populations that make studies from other regions inapplicable to the MENA. In spite of the multiethnic origins, high rates of consanguinity in the subpopulations render the genetic pool paradoxically limited and significantly increase not only the risk of congenital abnormalities but also the susceptibility of the population to chronic diseases and genetic disorders [167]. Screening for the common polymorphisms in the MENA can give insights on their prevalence in the region or can help discover polymorphisms indigenous for the region. Such action could help alleviate the burden of chronic diseases in the MENA simply by suggesting adequate adjustments of dietary factors to hide a genetic polymorphism or to prevent DNA damage.

 In some of the examples provided previously, success can be achieved, but in others, researchers ought to be more cautious. Selecting which polymorphisms are to be screened for, at the population level in the MENA, should be made after careful understanding of the effects of these variants on diet and disease. This is crucial to avoid misleading results and unnecessary costs. Unfortunately, functional studies are limited, but wide-scale screening and associations from other regions in the world could guide the decision-making process regarding screening in the MENA. At the same time, more effort and money should be invested in molecular and cellular research in nutritional genomics in order to better understand the function of the dietary signature and to more confidently guide population screening and personalized diet.

## 8. Conclusions

- MENA countries are witnessing a radical change in dietary patterns from a traditional diet to a less healthy industrialized diet.
- Rising prevalence for civilization diseases of metabolism and micronutrient deficiencies in the MENA parallels the change in dietary habits and is mostly caused by it.
- Nutrigenomic factors and the dietary signature on the genome play a role in the diet-disease interactions.
- Genetic sequence variations, epigenetic profiles, and posttranscriptional and posttranslational modifications are some of the mechanisms that define the diet-genetics-disease relationship.
- A large set of gene polymorphisms have been correlated with civilization diseases of metabolism, only a little of which have been studied in MENA countries.
- There are different mechanisms through which diet-genetics interaction affects micronutrient pathways and contributes to disease, including vitamin D and calcium, iron, folate, and vitamins C and E.
- Given the drastic dietary changes in the region over a short period of time, diet is the most obvious public health intervention, yet system biology and genomics research should not be underestimated.
- Wide-scale screening for certain gene polymorphisms in the MENA might allow for efficient intervention with personalized diet.
- More nutrigenomics research is needed to look at function and mechanisms of the diet-genetics-disease interaction.

## Supplementary Material

Supplementary Material: shows references for tables and figures.

## Figures and Tables

**Table 1:** Rates of cardiovascular disease, hypertension, and the metabolic syndrome in MENA countries from different studies.

Coronary artery disease (CAD)			

Iran	Age-adjusted prevalence (%)	12.7	Nabipour et al. [73]
Jordan	Prevalence (%)	5.9	Nsour et al. [74]

Saudi Arabia,			
(rural)	Prevalence (%)	4.0	Al-Nozha et al. [75]
(urban)	Prevalence (%)	6.2	
(overall)	Prevalence (%)	5.5	

Tunisia	Prevalence (%), [men]	12.5	Ben Romdhane et al. [76]
	Prevalence (%), [women]	20.6	

Cerebrovascular Accidents			

Bahrain	Age-adjusted incidence (per 100,000)	96.2	Al-Jishi and Mohan [77]
Iran	Age-adjusted incidence (per 100,000)	61.5	Ahangar et al. [78]
Kuwait	Age-adjusted incidence (per 100,000)	92.2	Abdul-Ghaffar et al. [79]
Libya	Age-adjusted incidence (per 100,000)	114.2	Radhakrishnan et al. [80]
Palestine	Age-adjusted incidence (per 100,000)	62.7	Sweileh et al. [81]
Qatar	Age-adjusted incidence (per 100,000)	123.7	Hamad et al. [82]
Saudi Arabia	Age-adjusted incidence (per 100,000)	38.5	Al-Rajeh et al. [83]

Hypertension (HTN)			

Algeria	Prevalence (%), [Age > 25]	36.2	Yahia-Berrouiguet et al. [84]
Bahrain	Prevalence (%), [Age > 20]	42.1	Al-Zurba [85]

Egypt	Age-adjusted prevalence (%)	27.4	Ibrahim et al. [86]
	Prevalence (%), [Age > 25]	26.3	Galal [87]

Iran	Prevalence (%), [Age > 19]	25.6	Sarraf-Zadegan et al. [88]
	Prevalence (%), [Age: 30–55]	23.0	Haghdoost et al. [89]
	Prevalence (%), [Age > 55]	49.5	

Iraq	Prevalence (%), [Age > 20]	19.3	WHO: STEPwise, [90]
Jordan	Prevalence (%), [Age > 18]	30.2	Zindah et al. [91]
Lebanon	Prevalence (%), [Age: 18–65]	31.2	Sibai et al. [92]
Morocco	Prevalence (%), [Age > 20]	33.6	Tazi et al. [93]
Oman	Prevalence (%), [Age > 20]	21.5	Hasab et al. [94]

Palestine (WB),			
(rural)	Prevalence (%), [Age: 30–65]	25.4	Abdul-Rahim et al. [95]
(urban)	Prevalence (%), [Age: 30–65]	21.5	

Qatar	Prevalence (%), [Age: 25–65]	32.1	Bener et al. [96]
Saudi Arabia	Prevalence (%), [Age: 30–70]	26.1	Al-Nozha et al. [97]
Sudan	Prevalence (%), [Age: 25–64]	23.6	WHO: STEPwise, [90]
Syria	Prevalence (%), [Age: 18–65]	40.6	Maziak et al. [98]
Turkey	Age-adjusted prevalence (%)	25.7	Sonmez et al. [99]
UAE	Prevalence (%), [Age > 20]	20.8	Baynouna et al. [100]
Yemen	Prevalence (%), [Age > 35]	26.0	Gunaid and Assabri [101]
Middle East	Prevalence (%), (overall)	21.7	Motlagh et al. [102]

Metabolic Syndrome			

Algeria	Prevalence (%), [Age > 20]	17.4	Mehio Sibai et al. [103]
Iran	Prevalence (%), [Age > 19]	23.3	Mehio Sibai et al. [103]
Jordan	Prevalence (%), [Age > 18]	36.3	Khader et al. [104]
Kuwait	Prevalence (%), [Age > 20]	24.8	Al Rashdan and Al Nesef [105]
Lebanon	Prevalence (%), [Age: 18–65]	25.4	Mehio Sibai et al. [103]
Morocco, (rural)	Prevalence (%), [women]	16.3	Rguibi and Belahsen [106]

Oman,			
(overall)	Prevalence (%), [Age > 20]	21.0	Al-Lawati et al. [107]
(Nizwa)	Age adjusted prevalence (%)	8.0	Al-Lawati et al. [107]

Palestine (WB)	Prevalence (%), [Age: 30–65]	17.0	Abdul-Rahim et al. [95]

Qatar	Prevalence (%), [Age > 20]	27.7	Musallam et al. [108]
	Age adjusted prevalence (%)	26.5	Bener et al. [109]

Saudi Arabia	Age adjusted prevalence (%)	39.3	Al-Nozha et al. [110]
Tunisia	Prevalence (%), [Age > 20]	16.3	Bouguerra et al. [111]
UAE	Prevalence (%)	39.6	Malik and Razig [112]

Nonadjusted rates from different studies are not valid for comparison but displayed to present the burden of the morbidities. HTN is defined as BP > 140/90 or use of antihypertensive medications. Metabolic Syndrome definition is based on Adult Treatment Panel III, except for Palestine and Tunisia where, respectively, WHO criteria and hypercholesterolemia (Total Cholesterol ≥5.2 mmol/l) instead of low HDL cholesterol were used. UAE: United Arab Emirates; WB: West Bank [103, 113–116].

**Table 2:** Rates of Vitamin D deficiency and iron deficiency in MENA countries from different studies.

Vitamin D deficiency (VDD)			

Iran	Prevalence (%), [girls], [adolescent]	Up to 70	Moussavi et al. [143]

Jordan	Prevalence (%),		
	[adult females]	37.3	Batieha et al. [144]
	[adult males]	5.1	

Lebanon	Prevalence (%),		
	[girls]	32	El-Hajj Fuleihan et al. [145, 146]
	[boys]	9–12	

Morocco (Rabat)	Prevalence (%), [women]	91	Arabi et al. [147]
Saudi Arabia	Prevalence (%), [girls], [adolescent]	Up to 80	Siddiqui and Kamfar [148]
Tunisia (Ariana)	Prevalence (%), [women], [Age: 20–60]	47.6	Arabi et al. [147]

Turkey (Ankara)	Prevalence (%),		
	[mothers]	46	Arabi et al. [147]
	[newborns]	80	

Iron deficiency			

Arab Gulf countries	Prevalence (%),		
	[children], [preschool age]	20–67	Musaiger [149]
	[children], [school age]	12.6–50	
	[pregnant women]	22.7–54	

Bahrain	Prevalence (%),		
	[children], [Age: 6–59 months]	48	Bagchi [150]
	[women], [Age: 15–49]	37.3	

Egypt	Prevalence (%),		
	[children], [Age: 6–59 months]	25	Bagchi [150]
	[women], [Age: 15–49]	11	

Iran	Prevalence (%),		
	[children], [Age: 6–59 months]	15–30	Bagchi [150]
	[women], [Age: 15–49]	33.4	

Jordan	Prevalence (%),[children], [school age]	20	Bagchi [150]
	Prevalence (%), [women], [Age: 15–49]	28	

Lebanon	Prevalence (%), [children], [Age: 6–59 months]	23	Bagchi [150]

Morocco	Prevalence (%),		
	[children], [Age: 6–59 months]	35	Bagchi [150]
	[women], [Age: 15–49]	30.1	

Oman	Prevalence (%),		
	[children], [Age: 5–14]	41	Bagchi [150]
	[women], [Age: 15–49]	40	

Pakistan	Prevalence (%),		
	[children], [Age: 6–59 months]	60	Bagchi [150]
	[women], [Age: 15–49]	30	

Palestine	Prevalence (%),		
	[children], [Age: 6–59 months]	53	Bagchi [150]
	[women], [Age: 15–49]	36.2	

Saudi Arabia	Prevalence (%), [children], [preschool age]	17	Bagchi [150]

Syria	Prevalence (%),		
	[children], [Age: 6–59 months]	23	Bagchi [150]
	[women], [Age: 15–49]	40.8	

UAE	Prevalence (%),		
	[children], [Age: 6–59 months]	34	Bagchi [150]
	[pregnant women]	14	

Yemen	Prevalence (%), [children], [preschool age]	70	Bagchi [150]

Different limits of blood levels define VDD, ranging from insufficiency to severe deficiency, similar for Iron deficiency. UAE: United Arab Emirates [147, 150].

## Abbreviations

MENA: Middle East and North Africa
WHO: World Health Organization
OMIM: Online Mendelian Inheritance in Man
NCBI: National Center for Biotechnology Information
SNP: Single Nucleotide Polymorphism
MNDs: Micronutrient Deficiencies.
