# Supplementary material for: Diet, Genetics, and Disease: A Focus on the Middle East and North Africa Region
Source: J Nutr Metab. 2012 Mar 29;2012:109037. doi: 10.1155/2012/109037 (PMC3321453; doi:10.1155/2012/109037)
Supplement: Supplementary file 1 — Supplementary Material: shows references for tables and figures. [file 109037.f1.pdf]

Online Supplement: References for Tables and Figures

- (Wild 2004) Wild S, Roglic G, Green A, Sicree R, King H. Global prevalence of diabetes: estimates for the year 2000 and projections for 2030. Diabetes Care. 2004 May;27(5):1047-53. PMID:15111519

- (Nabipour 2007) Nabipour I, Amiri M, Imami SR, et al. The metabolic syndrome and nonfatal ischemic heart disease; a population-based study. Int J Cardiol 2007;118:48-53.

- (Nsour 2008) Nsour M, Mahfoud Z, Kanaan MN, et al. Prevalence and predictors of nonfatal myocardial infarction in Jordan. East Mediterr Health J 2008;14:818-30.

- (Al-Nozha 2004) Al-Nozha MM, Arafah MR, Al-Mazrou YY, et al. Coronary artery disease in Saudi Arabia. Saudi Med J 2004;25:1165-71.

- (Romdhane 2002) Habiba Ben Romdhane, Raoudha Khaldi, AbdelmajidOueslati, Hajer Skhiri. Transition épidémiologique et transition alimentaire et nutritionnelle en Tunisie. Options Méditerranéennes, Sér. B / n°41, 2002 - La surveillance alimentaire et nutritionnelle en Tunisie

- (Al-Jishi 2000) Al-Jishi A, Mohan P. Profile of stroke in Bahrain. Neurosciences 2000;5(1):30-4.

- (Ahangar 2005) Ahangar AA, Ashraf Vaghefi SB, Ramaezani M. Epidemiological evaluation of stroke in Babol, northern Iran (2001–2003). Eur Neurol 2005;54(2):93-7.

- (Abdul-Ghaffar 1997) Abdul-Ghaffar NU, el-Sonbaty MR, el-Din Abdul-Baky MS, Marae AA, al-Said AM. Stroke in Kuwait: a three-year prospective study. Neuroepidemiology 1997;16 (1):40–7.

- (Radhakrishnan 1986) Radhakrishnan K, Ashok PP, Sridharan R, el-Mangoush MA. Incidence and pattern of cerebrovascular diseases in Benghazi, Libya. J Neuro Neurosurg Psychiatry 1986;49(5):519–23.

- (Sweileh 2008) Sweileh WM, Sawalha AF, Al-Aqad SM, Zyoud SH, Al-Jabi SW, Sweileh WM, et al. The epidemiology of stroke in northern palestine: a 1-year, hospital-based study. J Stroke Cerebrovasc Dis Nov–Dec 2008;17(6):406–11.

- (Hamad 2001) Hamad A, Sokarb TEO, Momeni S, Mesraoua B, Lingren A. Stroke in Qatar: a one-year hospital based study. J Stroke Cerebrovasc Dis 2001;10(5):236–41.

- (al-Rajeh 1998) al-Rajeh S, Larbi EB, Bademosi O, Awada A, Yousef A, al-Freihi H, et al. Stroke register: experience from the eastern province of Saudi Arabia. Cerebrovasc Dis 1998;8(2):86–9.

- (Yahia-Beerouiguet 2009) Yahia-Beerouiguet A, Benyoucef M, Meguenni K, Brouri M: Prevalence of cardiovascular risk factors: a survey at Tlemcen (Algeria). Med Mal Metab 2009; 3:313–319.

- (Al-Zurba 2001) Al-Zurba FI: Latest studies clarify state of health in Bahrain. Diabetes Voice 2001; 46:28–31.

- (Ibrahim 1995) Ibrahim MM, Rizk H, Appel LJ, Aroussy W, Helmy S, Sharaf Y, et al. Hypertension prevalence, awareness, treatment, and control in Egypt. Results from the Egyptian National Hypertension Project (NHP). NHP Investigative Team. Hypertension 1995; 26:886 – 890.

- (Galal 2002) Galal O: The nutrition transition in Egypt: obesity, undernutrition and the food consumption context. Public Health Nutr 2002; 5:141–148.

- (Sarraf-Zadegan 1999) Sarraf-Zadegan N, Boshtam M, Mostafavi S, Rafiei M. Prevalence of hypertension and associated risk factors in Isfahan, Islamic Republic of Iran. East Mediterr Health J 1999; 5:992 – 1001.

- (Haghdoost 2008) Ali-Akbar Haghdoost, Behnam Sadeghirad, Mohammad Rezazadehkermani. Epidemiology and Heterogeneity of Hypertension in Iran: A Systematic Review. Arch Iranian Med 2008; 11 (4): 444 – 452

- (WHO: STEPwise 2007) World Health Organization: STEPwise surveillance. Non-communicable diseases risk factors. STEPwise data from selected countries in the Eastern Mediterranean Region, 2003–2007. http://www.emro.who.int/ncd/risk_factors.htm#physical (accessed October 2009).

- (Zindah 2008) Zindah M, Belbeisi A, Walke H: Obesity and diabetes in Jordan: findings from the Behavioral Risk Factor Surveillance System. Prev Chronic Dis Public Health Res Pract Policy 2008; 5: 1–8.

- (Sibai 2008) Sibai A, Obeid O, Batal M, et al: Prevalence of metabolic syndrome in Lebanese adult population: findings from the first epidemiological 83–90.

- (Tazi 2003) Tazi MA, Abir-Khalil S, Chaouki N, Cherqaoui S, Lahmouz F, Sraïri JE, Mahjour J: Prevalence of the main cardiovascular risk factors in Morocco: results of a national survey 2000. J Hypertens 2003; 21:897–903.

- (Hasab 1999) Hasab AA, Jaffer A, Hallaj Z: Blood pressure patterns among the Omani population. East Mediterr Health J 1999; 5: 46–54.

- (Abdul-Rahim 2003) Abdul-Rahim HF, Holmboe-Ottesen G, Stene LC, Husseini A, Giacaman R, Jervell J, Bjertness E: Obesity in a rural and an urban Palestinian West Bank population. Int J Obes Relat Metab Disord 2003; 27: 140–146.

- (Bener 2004) Bener A: The prevalence of hypertension and its associated risk factors in a newly developed country. Saudi Med J 2004; 25: 918–922.

- (Al-Nozha 2007) Al-Nozha MM, Abdullah M, Arafah MR, Khalil MZ, Khan NB, Al-Mazrou YY, Al-Maatouq MA, Al-Marzouki K, Al-Khadra A, Nouh MS, Al-Harthi SS, Al-Shahid MS, Al-Mobeireek A: Hypertension in Saudi Arabia. Saudi Med J 2007; 28:77–84.

- (Maziak 2007) Maziak W, Rastam S, Mzayek F, Ward KD, Eissenberg T, Keil U: Cardiovascular health among adults in Syria: a model from developing countries. Ann Epidemiol 2007; 17: 713–720.

- (Sonmez 1999) Sonmez HM, Basak O, Camci C, Baltaci R, Karazeybek HS, Yazgan F, et al. The epidemiology of elevated blood pressure as an estimate for hypertension in Aydin, Turkey. J Hum Hypertens 1999; 13:399 – 404.

- (Baynouna 2008) Baynouna LM, Revel AD, Nagelkerke NJ, Jaber TM, Omar AO, Ahmed NM, Naziruldeen MK, Al-Sayed MF, Nour FA: High prevalence of the cardiovascular risk factors in Al-Ain, United Arab Emirates: an emerging health care priority. Saudi Med J 2008; 29:1173–1178.

- (Gunaid 2008) Gunaid AA, Assabri AM: Prevalence of type 2 diabetes and other cardiovascular risk factors in a semi-rural area in Yemen. Rev Santé Méditerr Orientale 2008; 14:42–56.

- (Motlagh 2009) Motlagh B, O'Donnell M, Yusuf S. Prevalence of cardiovascular risk factors in the Middle East: a systematic review. European Journal of Cardiovascular Prevention & Rehabilitation. 16(3):268-280, June 2009.

- (Khader 2007) Khader Y, Bateiha A, El-Khateeb M, Al-Shaikh A, Ajlouni K: High prevalence of the metabolic syndrome among Northern Jordanians. J Diabetes Complications 2007; 21:214–219.

- (Al Rashdan 2009) Al Rashdan IR, Nesef YA. Prevalence of overweight, obesity and metabolic syndrome among adult Kuwaitis: results from community-based national survey. Angiology 2010; 61: 42–48.

- (Rguibi 2004) Rguibi M, Belahsen R: Metabolic syndrome among Moroccan Sahraoui adult women. Am J Hum Biol 2004; 16: 598–601.

- (Al-Lawati 2003) Al-Lawati JA, Mohammed AJ, Al-Hinai HQ, Jousilahti P: Prevalence of the metabolic syndrome among Omani adults. Diabetes Care 2003; 26: 1781–1785.

- (Al-Lawati 2006) Al-Lawati JA, Jousilahti P. Prevalence of metabolic syndrome in Oman using the International Diabetes Federation’s criteria. Saudi Med J 2006; 27: 1925–1926.

- (Musallam 2008) Musallam M, Bener A, Zirie M, Al-Gaud YK, Al-Hamaq A, Othman M, Tewfik I: Metabolic syndrome and its components among Qatari population. Int J Food Safety Nutr Public Health 2008; 1: 88–102. 1044.

- (Bener 2009) Bener A, Zirie M, Musallam M, Khader YS, Al-Hamaq AO. Prevalence of metabolic syndrome according to Adult Treatment Panel III and International Diabetes Federation criteria: a population-based study. Metab Syndr Relat Disord 2009; 7: 221–229.

- (Al-Nozha 2005) Al-Nozha M, Al-Khadra A, Arafah MR, Al-Maatouq MA, Khalil MZ, Khan NB et al. Metabolic syndrome in Saudi Arabia. Saudi Med J 2005; 26: 1918–1925.

- (Bouguerra 2006) Bouguerra R, Ben Salem L, Alberti H, Ben Rayana C, El Atti J, Blouza S, Gaigi S, Achour A, Ben Slama C, Zouari B: Prevalence of metabolic abnormalities in the Tunisian adults: a population based study. Diabetes Metab 2006; 32: 215–221.

- (Malik 2008) Malik M, Razig SA. The prevalence of the metabolic syndrome among the multiethnic population of the United Arab Emirates: a report of a national survey. Metab Syndr Relat Disord 2008; 6:177–186.

- (Kearney 2004) Kearney PM, Whelton M, Reynolds K, Whelton PK, He J. Worldwide prevalence of hypertension: a systematic review. Journal of Hypertension 2004, 22:11 – 19

- (Sibai 2010) Abla Mehio Sibai, Lara Nasreddine, Ali H. Mokdad, Nada Adra, Maya Tabet, Nahla Hwalla. Nutrition Transition and Cardiovascular Disease Risk Factors in Middle East and North Africa Countries: Reviewing the Evidence. Ann Nutr Metab 2010;57:193–203

- (Mabry 2010) R. M. Mabry, M. M. Reeves, E. G. Eakin, N. Owen. Gender differences in prevalence of the metabolic syndrome in Gulf Cooperation Council Countries: a systematic review. Diabet. Med. 27, 593–597 (2010)

- (Gaziano 2010) Gaziano TA, Bitton A, Anand S Abrahams-Gessel S, Murphy A. Growing Epidemic of Coronary Heart Disease in Low- and Middle-Income Countries. Curr Probl Cardiol 2010;35:72-115.

- (Tran 2010) Jackie Tran, Masoud Mirzaei, Laurie Anderson, Stephen R. Leeder. The epidemiology of stroke in the Middle East and North Africa. Journal of the Neurological Sciences 295 (2010) 38–40

- (IDF 2000) International Diabetes Federation, Diabetes Atlas. Ed.1 (2000) www.eatlas.idf.org Accessed July 11, 2011

- (IDF 2003) International Diabetes Federation, Diabetes Atlas. Ed.2 (2003) www.eatlas.idf.org Accessed July 15, 2011

- (IDF 2007) International Diabetes Federation, Diabetes Atlas. Ed.3 (2007) www.eatlas.idf.org Accessed July 11, 2011

- (IDF 2010) International Diabetes Federation, Diabetes Atlas. Ed.4 (2010) www.eatlas.idf.org Accessed June 27, 2011

- (WHS 2005) World Health Statistics Annual 2005. www.who.int/whosis/whostat Accessed June 25, 2011

- (WHS 2011) World Health Statistics Annual 2011. www.who.int/whosis/whostat Accessed June 25, 2011

- (Yusuf 2001) Salim Yusuf, Srinath Reddy, Stephanie Ôunpuu, Sonia Anand. Global Burden of Cardiovascular Diseases: Part I: General Considerations, the Epidemiologic Transition, Risk Factors, and Impact of Urbanization. Circulation 2001;104;2746-2753

- (Bagchi, 2004) Iron deficiency anaemia – an old enemy, K. Bagchi, La Revue de Santé de la Méditerranée orientale Volume 10, No. 6, November 2004, Pages 754 – 760

- (Musaiger , 2002) Iron deficiency anaemia among children and pregnant women in the Arab Gulf countries: the need for action, Musaiger AO., Nutr Health. 2002;16(3):161-71.

- (Arabi, 2010), Hypovitaminosis D in developing countries prevalence, risk factors and outcomes, Asma Arabi, Rola El Rassi and Ghada El-Hajj Fuleihan, OCTOBER 2010, VolumE 6,

- (Siddiqui , 2007) Siddiqui AM, Kamfar HZ (2007) Prevalence of vitamin D deficiency rickets in adolescent school girls in Western region, Saudi Arabia. Saudi Med J 28:441-444.

- (El-Hajj Fuleihan ,2001) El-Hajj Fuleihan G, Nabulsi M, Choucair M, et al. (2001)

Hypovitaminosis D in healthy schoolchildren. Pediatrics 107:E53.

- (El-Hajj Fuleihan , 2006) El-Hajj Fuleihan G, Nabulsi M, Tamim H, et al. (2006)

Effect of vitamin D replacement on musculoskeletal parameters in school children: a

randomized controlled trial. J Clin Endocrinol Metab 91:405-412.

- (Moussavi , 2005) Moussavi M, Heidarpour R, Aminorroaya A, et al. (2005) Prevalence of vitamin D deficiency in Isfahani High School students in 2004. Horm Res. 2005;64(3):144-8. Epub 2005 Sep 27.

- (Batieha, 2011) Vitamin D Status in Jordan: Dress Style and Gender Discrepancies A. Batieha, Y. Khader, H. Jaddou, D. Hyassat, Z. Batieha, M. Khateeb, A. Belbisi, K. Ajlouni, Ann Nutr Metab 2011;58:10-18
